# Parents' intention for their children to receive COVID-19 vaccine: Implications for vaccination program in Macao

**DOI:** 10.3389/fped.2022.978661

**Published:** 2022-10-03

**Authors:** Un I Choi, Yimin Pang, Yu Zheng, Pou Kuan Tang, Hao Hu, Carolina Oi Lam Ung

**Affiliations:** ^1^State Key Laboratory of Quality Research in Chinese Medicine, Institute of Chinese Medical Science, University of Macau, Zhuhai, Macao SAR, China; ^2^Department of Public Health and Medicinal Administration, Faculty of Health Sciences, University of Macau, Zhuhai, Macao SAR, China

**Keywords:** parent, intention, children, COVID-19, vaccine, Macao (Macau), SARS-CoV-2

## Abstract

**Introduction:**

The decision about vaccinating children is subject to their parents' decision. To inform strategies that support full vaccination coverage, it is important to understand the parents' vaccination attitude and tendency to act. This study aims to investigate the intention and the factors affecting parents' decision-making about vaccinating their children.

**Methods:**

A cross-sectional, self-administered online questionnaire was completed by parents of children aged 3–12 yeas in Macao between 7 March and 17 April 2022. The survey tool was informed by the Theory of Planned Behavior (TPB) which composes of the variable “intention” and three TPB constructs (*Attitude, Subjective Norm*, and *Perceived Behavioral Control*). Respondents rated their level of agreement on the construct statements using a 5-point Likert scale. Multiple linear regression analysis was used to determine if the TPB constructs were predictors of parents' intention.

**Results:**

A total of 1,217 parents completed the questionnaire. The majority of participants were mothers (83.2%), aged 31–40 years (62.7%), having two or more children (74.1%), had at least one dose of COVID-19 vaccine (84.4%) and considered themselves knowledgeable about the vaccine (62.1%), all of which were significantly associated with the intention to vaccinate their children (all *p* < 0.05). Their intention varied from negative (19.1%), neutral (38.4%) to positive (42.5%). Respondents were mostly concerned about the serious side effects that the COVID-19 vaccine (mean = 3.96 ± 1.23), highly acknowledged the expectation by the school (mean = 3.94 ± 1.15) and the community (mean = 3.90 ± 1.19) of children vaccination, and rated highly the ease of making necessary arrangement (mean = 3.93 ± 1.25). In the multiple linear regression model which explained 63.5% of the variance in the intention-to-vaccinate their children, only *Attitude* (B = 0.52, *p* < 0.001) and *Subjective Norm* (B = 0.39, *p* < 0.001) were identified as strong predictors. The major reasons for not having intention were safety concerns (*n* = 646/699, 92.4%). Participants' most trusted local information sources were doctors (*n* = 682), government (*n* = 426) and healthcare professional organizations (*n* = 416).

**Conclusions:**

Vaccinating children with COVID-19 vaccine is a complex decision-making for parents. A key to a successful COVID-19 vaccination program is effective communication about the safety profile and the usage experiences warranting the integration of reliable information sources across different healthcare sectors.

## Introduction

COVID-19 vaccination for children entails both individual benefits (protecting them from rare but severe cases of COVID-19 infection) and community benefits (protecting others by reducing the spread of the virus) (1). Although the risk of severe illness and death from the COVID-19 remains quite low for children, more cases and more serious symptoms among younger children have been linked to the emergence of new variants (2). Reportedly, the incidence rate of the Omicron variant was 6–8 times that of the Delta variant in children younger than 5 years (3, 4). Infected children may be at risk of severe illnesses such as croup and multisystem inflammatory syndrome requiring more aggressive treatment, hospitalization or even intensive care unit admission (5, 6) in addition to long-term symptoms (7). Although not common, COVID-19 deaths did occur among children. According to the UNICEF, as of March 2022, of the 13,400 COVID-19 deaths in people under 20 years of age reported, 58% occurred among people aged 10–19 and 42% among children aged 0–9 (8). Vaccination against COVID-19 remains one of the most effective protection against infection. Moreover, high vaccination coverage among children is also crucial to achieving herd immunity for the overall control of the COVID-19 pandemic (9–11).

For children under the age of 18 years, parents are usually the key, if not the sole, decision-makers for whether they will receive a COVID-19 vaccine. To facilitate the uptake of COVID-19 among children, it is important to understand parents' acceptability of their children's COVID-19 vaccination and related barriers and facilitators. However, the current research shows that parents' intentions and refusal and the influencing factors vary substantially between studies. For instance, a systematic review involving 44 studies including 317,055 parents found that the overall proportion of parents that intend to vaccinate their children against COVID-19 was 60.1%, but the heterogeneity ranged from 25.6 to 92.2% (12). Similar variations were also reported in another systematic review involving 29 studies from 16 countries and regions with 68,327 participants (13), which found that the vaccination willingness could be as high as 91.4% (14), or as low as 21.6% (15).

Factors influencing the decision about COVID-19 vaccination were multifaceted and might include sociodemographic characteristics, attitudes toward vaccination, psychological factors, perceptions of risk and susceptibility to COVID-19, knowledge, information, personal factors, etc. (16–18). While being fathers, older age of parents, older age of children, higher income, and higher levels of perceived threat from the COVID-19 had been commonly identified as the predictors (12, 13), how such factors influenced parents' intention remained inconclusive. Parents' intention may also be influenced by vaccine hesitancy constituted by concerns about vaccine safety and effectiveness and variations in their information accessibility and sources (19–21). As parents' intentions and the predictors are context-specific, research specific to the local context is needed to inform vaccination programs more precisely (12).

Currently, no existing scale is available to assess parents' expressed intent to have their children receive COVID-19 vaccines. A theoretically informed approach was employed in order to provide a foundation to gather, interpret, and analyze data in a systematic manner in this study (22). The Theory of Planned Behavior (TPB) being one of the most robust models for explaining health-related behavior has been widely used as a practical framework to understand important factors for intention to uptake vaccination, including COVID-19 vaccines (23, 24) as well as influenza and human papillomavirus (HPV) (25–27). In light of the ongoing need to investigate parents' intention to have their children receive COVID-19 vaccines and the possible influencing factors, particularly in areas whereby intention may be negatively affected by low incidence of COVID-19 cases, this study aimed to (1) investigate the parents' intention to have their children receive COVID-19 vaccines by employing the TPC model; (2) to identify the main factors predicting their intention; and (3) to assess the usefulness of the TPB model in predicting such intention. Understanding parental COVID-19 intention for the children and the factors affecting their decision-making would help inform evidence-based actions that change the stereotypes and to facilitate the success of COVID-19 vaccination program.

## Methods

This study employed a cross-sectional survey informed by the TPB framework and self-administered online voluntarily by parents of children aged between 3 and 12 years old in Macao between 7 March and 17 April 2022. The survey was only open for 6 weeks in order to provide a snapshot of the parents' intention soon after the government lowered the age limit for the COVID-19 vaccination down to 12 years of age (previously only available to people over 16 years old).

The project was approved by the Panel on Research Ethics of the University of Macau in January 2022 (SSHRE21-APP064-ICMS). As indicated in the Participant Information Statement (PIS), it was assumed that, by completing and submitting the survey online, they agreed to take part in the research study. At the beginning of the survey, a checkbox was set for the respondents to clearly indicate their consent before proceeding to answer the survey questions. The PIS also clearly indicated the study purpose, the potential use of the information provided by the respondents and the measures in place to protect their confidentiality. No incentives were offered to the respondents upon completion of the survey. Following the Strengthening the Reporting of Observational Studies in Epidemiology (STROBE) guideline and the Checklist for Reporting Results of Internet E-Surveys (CHERRIES) (28, 29), the reporting of the study is as follows.

### Study target

Macao is one of the most densely populated places in the world with a population of 682,800 (in the third quarter of 2020) and is a famous tourist destination, exposing the city to a high risk of community transmission and imported cases amid the COVID-19 pandemic. As of February 21, 2022, the city had 80 cumulative confirmed cases of COVID-19, with the first case confirmed on January 22, 2020, all of those having recovered from the disease (30). The top-down infection control actions undertaken by the local government have been effective in keeping the infection rate low and preventing community transmission in the city. Macao remained as one of the very few “COVID zero” regions across the globe at the time of this study (30, 31).

On February 9, 2021, the Macao government launched the COVID-19 vaccination campaign to provide free COVID-19 vaccines to all Macao residents and people who were studying or working in Macao. The supply of COVID-19 vaccines had been steady, an online registration system had been established, and the vaccination sites had been widely distributed to be in close proximity to the people's neighborhood. A 1-year group insurance for COVID-19 vaccine recipients was also arranged to cover major adverse events associated with vaccination. Information about the doses of COVID-19 vaccines given and adverse events reported was updated on the Health Bureau official website on a daily basis. In November 2021, the government lowered the age limit for the COVID-19 vaccination down to 3 years of age. Since then, the uptake of vaccines among young children has been slow. As of February 21, 2022, the vaccination rate among children aged 3–11 years old was only 8.5%, whereas that for the age groups of 12–19 years and 20–49 years were 72.9% and well over 96%, respectively (32). By law, parents need to give consent before people under 18 years of age can receive COVID-19 vaccines.

The target population of this survey study was parents or legal guardians of people aged between 3 and 12 years residing in Macao during the COVID-19 pandemic. According to the official statistics, there were around 115,600 people aged 18 or younger in Macao as of 2019. Assuming they are all the only children, the maximum number of parents eligible for this study would be 231,200 (115,600 × 2). Based on a sample population of 231,200, the valid sample size is determined at a minimum of 384 (confidence level 95%, margin of error 5%).

### Questionnaire design

The design of the questionnaire was developed in consultation with: (1) the current literature on parents' intention to get their children vaccinated with COVID-19 vaccines and other vaccines (26, 33–35), (2) studies employing the Theory of Planned Behavior as the theoretical framework (36, 37), and (3) a clinician experienced in public health measures and the local COVID-19 vaccination campaign.

**Figure 1:**
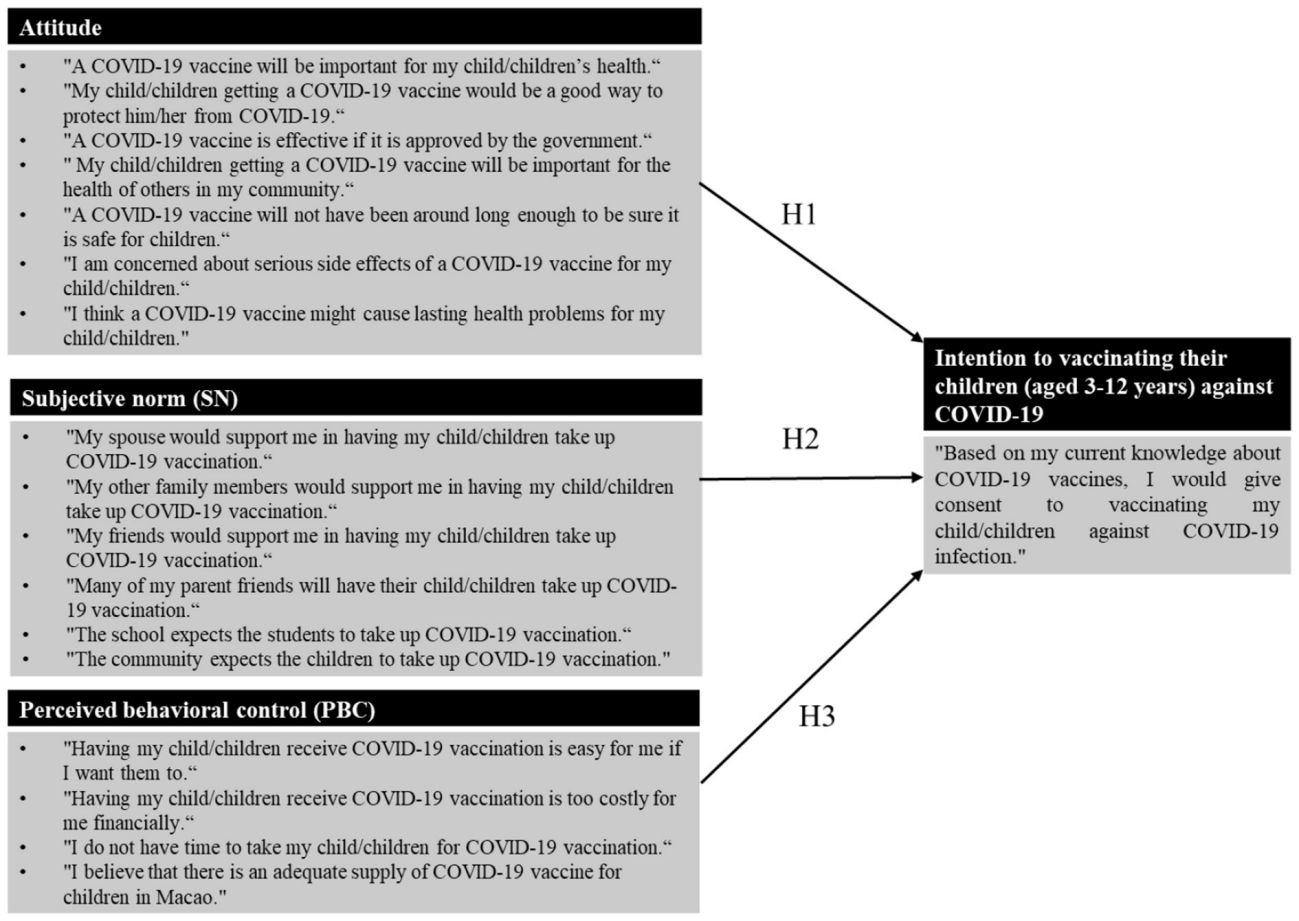
Research model of parents' intention to vaccinating their children (aged 3–12 years) against COVID-19.

According to the TPB, *Attitude, Subjective Norm*, and *Perceived Behavioral Control* are the key variables, which influence behavior through their impact on behavioral intention. As depicted in Figure 1, regarding the 3 constructs in the TPB model employed in this study, *Attitude* referred to the degree to which a parent had a favorable or unfavorable evaluation of the COVID-19 vaccine for the health of their children, *Subjective Norm* referred to the belief about whether peers and people of importance to the parents thought he or she should get their children vaccinated with COVID-19 vaccines, and *Perceived Behavior Control* referred to the perception of the parent on the level of ease or difficulty of having their children receive the vaccination.

The study hypotheses are, therefore, as follows:

H1: Favorable *Attitude* is a positive and significant predictor of the intention to have their children receive COVID-19 vaccines.

H2: Positive *Subjective Norm* is a positive and significant predictor of intention to have their children receive COVID-19 vaccines.

H3: Strong *Perceived Behavior Control* is a positive and significant predictor of intention to have their children receive COVID-19 vaccines.

The questionnaire mainly comprised of three sections. In Section A, the participants were asked to confirm if they were parents of children aged between 3 and 12 years residing in Macao and to answer 11 questions regarding their demographic information (including relationship to the child in question (38–40), age (41–43), marital status, number of children in total (14, 43), age of children in question (43, 44), highest education level attained (45, 46), type of residency, employment status, average monthly household income (47, 48), the personal history of COVID-19 vaccination (44, 49), and their perceived level of knowledge of COVID-19 vaccine (33, 45).

In Section B, there were 4 sub-sections, each of which contained a set of statements that measured *Attitude* (7 statements), *Subjective Norm* (6 statements), *Perceived Behavioral Control* (4 statements), and *Intention* (1 statement). Respondents were asked to rate their level of agreement on these items using a 5-point Likert scale with possible answers being strongly disagree, disagree, neutral, agree, and strongly agree.

In Section C, respondents were asked to identify factors hindering their decision to vaccinate their children and the information sources about COVID-19 vaccination among children deemed reliable. All the questions were assigned to be mandatory answer items to avoid incompleteness and missing data. At the conclusion of the questionnaire, a free-text response box was provided for respondents to provide additional feedback, and to indicate their interests in participating in a follow-up qualitative study to provide more in-depth insights about this topic.

### Development of the questionnaire

The self-administered structured questionnaire used in this study was prepared in English and Chinese in order to minimize sampling bias due to language barrier. The questionnaire underwent two rounds of pilot studies following the suggestions by Leavy (50). To ensure the face validity of the questionnaire that the theoretical constructs (*Attitude, Subjective Norm*, and *Perceived Behavioral Control* and *Intention*) were appropriately represented, the initial instrument was first assessed by three researchers experienced in quantitative studies and public health measures of mass vaccination through a focus group. The researchers thoroughly evaluate the statements and agreed that the measuring statements matched the corresponding constructs and collectively formed a valid measure of the TPB concept that addressed the research objectives. The researchers also provided minor suggestions on the wordings of the statement to improve the validity of the questionnaire design. All comments were taken into consideration.

The revised questionnaire was pilot tested on a convenience sample of 15 parents who were in bilingual (English and Chinese) and were invited through the authors' personal network. They were requested to specifically evaluate the content validity, content consistency and the time needed to complete. They all agreed that the questions were straightforward and easy to understand, confirming the face and content validity of the questionnaire. No removal of the original statements or addition of new ones was needed. None of the responses collected in the pilot tests was included in the study results for analysis.

### Data collection

The online questionnaire, hosted by Survey Monkey, was open for 6 weeks between 7 March and 17 April 2022. In order to minimize selection bias, multiple avenues were attempted to recruit respondents with diverse range of demographic characteristics: parents' communication groups, schools and social media (such as Facebook and WeChat). Invitations to participate in the study were sent to parents' communication groups of 20 primary schools through convenience sampling. Invitations to support the study were also extended to the schools and the link to the online survey was displayed during information session on COVID-19 vaccination hosted for parents at school. Invitations were also distributed through social media like Facebook and WeChat. Follow-up reminders were made in the parents' communication groups and on social media at two weekly intervals during the study period. A snowball sampling technique was also used whereby the participants were invited to pass the invitation and the survey link to their contacts. Given the wide usage of social media among the Macao population and the challenges of collecting data face-to-face during the pandemic, social media and online communication platform were chosen. To ensure the completeness of the answers, a logic function available was adopted to require an answer to every question before proceeding to the next page and before submission. Respondents were able to review and change their answers using a Back button before the submission of the survey, and were allowed to pause and continue answering the survey as long as the survey remained open. The survey was estimated to take around 6–8 min to complete. To minimize the risks of double entries or duplication of entries by the pharmacists, the setting allowing only one attempt per device was employed.

### Data analysis

The survey responses were analyzed using the Statistical Package for Social Sciences (SPSS) version 27 software for Windows. For respondents' demographic characteristics, descriptive analysis (frequencies) were conducted and univariate analysis using Pearson chi-square test was employed to compare the differences in the intention to get their children vaccinated with COVID-19 vaccines among subgroups. For the ratings given by the respondents' on TPB constructs and the sub-items, descriptive statistics (frequencies, means, and standard deviations) were conducted and Spearman's rho was used to test the correlation of intention with the constructs and the sub-items. Since the instrument was self-developed, Cronbach's alpha coefficients were used to determine the internal consistency of the measuring items for each construct.

Multiple linear regression analysis was conducted on the data to identify predictors of intention, with intention as the outcome factor and Attitude, Subjective Norm and Perceived Behavioral Control as the predictor factors. Demographic variables with a *p* < 0.05 in univariate analysis were also included in multivariable analysis. The demographic variables significant in univariate analysis were entered into the multivariable linear regression first, then the TPB construct variables were sequentially tested and retained when they remained statistically significant and contribute for a better fit of the model. All analyses based on two-sided *p*-values, and the association would be considered statistically significant at a confidence level of 95% whenever the *P*-value was found to be smaller than 0.05.

## Results

A total of 1,217 responses had been received, all of which were complete giving a completion rate of 100%. As shown in Table 1, the majority of the respondents were mothers (*n* = 1,013, 83.2%), aged between 31 and 40 years (*n* = 763, 62.7%) and had 2 children (*n* = 705, 57.9%). The age of their children in question ranged from 3 to 12 years of age. Around two-thirds of them (61.0%) had a Bachelor degree, 879 (72.2%) were employees, and 626 (51.5%) had a monthly household income of MOP50,000 or less (average monthly household income was MOP55,497 as of 2017) (the exchange rate of 1 USD was around 8.10 MOP) (51). Nearly 85% of the respondents had received COVID-19 vaccines themselves [45 (3.7%) had 1 dose, 740 (60.8%) had 2 doses, and 242 (19.9%) had 3 doses]. Most of them believed they were knowledgeable about the COVID-19 vaccines (*n* = 755, 62.1%).

**Table 1:** Respondents' demographic information (*n* = 1,217).

**Demographic information**		**Cases (*****n*** = **1,217)**		**Intention**						**X^2^**	***P***
		***n***	**%**	**No** **(*****n*** = **232, 19.1%)**		**Not sure** **(*****n*** = **467, 38.4%)**		**Yes** **(*****n*** = **518, 42.5%)**	
				**n**	**%**	**n**	**%**	**n**	**%**	
Relationship with the child in question	Father	193	15.9%	32	16.6%	55	28.5%	106	54.9%	−0.081**	0.005
	Mother	1,013	83.2%	197	19.4%	410	40.5%	406	40.1%		
	Legal guardian	11	0.9%	3	27.3%	2	18.2%	6	54.5%		
Age (years)	< 30	76	6.2%	22	28.9%	37	48.7%	17	22.4%	0.242**	0.000
	31–40	763	62.7%	172	22.5%	315	41.3%	276	36.2%		
	41–50	364	29.9%	37	10.2%	114	31.3%	213	58.5%		
	>50	14	1.2%	1	7.1%	1	7.1%	12	85.7%		
Marital status	Married	1,138	93.5%	216	19.0%	429	37.7%	493	43.3%	−0.019	*0.517*
	Cohabitant	18	1.5%	4	22.2%	13	72.2%	1	5.6%		
	Single	17	1.4%	6	35.3%	5	29.4%	6	35.3%		
	Widowed or divorced	37	3.0%	6	16.2%	14	37.8%	17	45.9%		
	Other	7	0.6%	0	0.0%	6	85.7%	1	14.3%		
Number of children	1	315	25.9%	78	24.8%	131	41.6%	106	33.7%	0.111**	0.000
	2	705	57.9%	117	16.6%	269	38.2%	319	45.2%		
	3	178	14.6%	37	20.8%	64	36.0%	77	43.3%		
	4	14	1.2%	0	0.0%	2	14.3%	12	85.7%		
	5 or more	5	0.4%	0	0.0%	1	20.0%	4	80.0%		
Age of the child in question (years)	3	180	14.8%	39	21.7%	90	50.0%	51	28.3%	0.220**	0.000
	4	124	10.2%	35	28.2%	61	49.2%	28	22.6%		
	5	137	11.3%	30	21.9%	56	40.9%	51	37.2%		
	6	156	12.8%	35	22.4%	59	37.8%	62	39.7%		
	7	150	12.3%	36	24.0%	47	31.3%	67	44.7%		
	8	119	9.8%	18	15.1%	48	40.3%	53	44.5%		
	9	105	8.6%	7	6.7%	37	35.2%	61	58.1%		
	10	88	7.2%	12	13.6%	25	28.4%	51	58.0%		
	11	85	7.0%	12	14.1%	24	28.2%	49	57.6%		
	12	73	6.0%	8	11.0%	20	27.4%	45	61.6%		
Highest education level attained	Secondary education or below	211	17.3%	29	13.7%	77	36.5%	105	49.8%	0.013	*0.643*
	Bachelor degree	742	61.0%	165	22.2%	298	40.2%	279	37.6%		
	Master degree or above	264	21.7%	38	14.4%	92	34.8%	134	50.8%		
Type of residency	Macao residents	1,186	97.5%	226	19.1%	457	38.5%	503	42.4%	0.012	*0.677*
	Non-Macao residents	31	2.5%	6	19.4%	10	32.3%	15	48.4%		
Employment status	Self employed	177	14.5%	50	28.2%	52	29.4%	75	42.4%	0.037	*0.197*
	Employee	879	72.2%	151	17.2%	355	40.4%	373	42.4%		
	Not employed	161	13.2%	31	19.3%	60	37.3%	70	43.5%		
Household income/per month (1USD = 8.10MOP)	< MOP15,000	75	6.2%	14	18.7%	31	41.3%	30	40.0%	0.031	*0.272*
	MOP15,001–30,000	223	18.3%	48	21.5%	87	39.0%	88	39.5%		
	MOP30,001–50,000	328	27.0%	63	19.2%	115	35.1%	150	45.7%		
	MOP50,001–80,000	390	32.0%	72	18.5%	161	41.3%	157	40.3%		
	MOP90,001–100,000	111	9.1%	21	18.9%	38	34.2%	52	46.8%		
	>MOP100,001	90	7.4%	14	15.6%	35	38.9%	41	45.6%		
The number of COVID-19 vaccine doses I have received	One dose	45	3.7%	13	28.9%	18	40.0%	14	31.1%	−0.061**	0.032
	Two doses	740	60.8%	129	17.4%	330	44.6%	281	38.0%		
	Three doses	242	19.9%	10	4.1%	48	19.8%	184	76.0%		
	Never	190	15.6%	80	42.1%	71	37.4%	39	20.5%		
Perceived knowledge of COVID-19 vaccine	Completely unclear	22	1.8%	7	31.8%	7	31.8%	8	36.4%	0.067**	0.019
	Unclear	78	6.4%	16	20.5%	39	50.0%	23	29.5%		
	Not sure	362	29.7%	58	16.0%	187	51.7%	117	32.3%		
	Clear	625	51.4%	110	17.6%	206	33.0%	309	49.4%		
	Completely clear	130	10.7%	41	31.5%	28	21.5%	61	46.9%		

^**^p < 0.01.

### Intention for their children to receive COVID-19 vaccines

Overall, 518 or 42.5% of the respondents indicated their intention for their children to receive COVID-19 vaccines, while 467 (38.4%) were hesitant and 322 (19.1%) did not want their children to get vaccinated. Among the subgroup, respondents who were fathers, aged 41 years or above, had more children, decided for older children, had a history of COVID-19 vaccination, or were perceived to have good knowledge of COVID-19 vaccines had a higher intention for their children to receive COVID-19 vaccines. According to the Pearson Chi-square test results, statistically significant differences in the intention were observed among the subgroups of the respondents' parental role, their age, the number of children they had, the age of the child in question, their personal history of COVID-19 vaccination, and their perceived knowledge of COVID-19 vaccines.

### Respondents' perception about the measurements

The ratings of individual survey statements under each of the three key constructs (*Attitude, Subjective Norm*, and *Perceived Behavioral Control*) and the intention are presented together with the percentage of respondents giving positive (strongly agree/agree), neutral, and negative (strongly disagree/disagree) responses. Descriptive statistics, such as the mean and standard deviation, of the ratings for each statement for each TPB key variable and intention were also listed.

In terms of the construct *Attitude*, the respondents agreed to the greatest extent that they were concerned about the serious side effects that the COVID-19 vaccine might cause to their children (mean = 3.96 ± 1.23). At the same time, the respondents also acknowledged to a high extent that getting their children to receive the COVID-19 vaccine would be important for the health of others in their community (mean = 3.90 ± 1.15), even more important than for their own child/children's health (mean = 3.45 ± 1.26). They were less likely to agree that the COVID-19 vaccine was effective just because it had been approved by the government (mean = 3.26 ± 1.25) which could be explained by their high rating for the statement about the COVID-19 vaccine being too new to fully understand its safety (mean = 3.77 ± 1.22). Almost half of the respondents (49%) believed that the COVID-19 vaccine might cause lasting health problems for their children, and 31.5% were not sure about it.

In terms of the construct *Subjective Norm*, the respondents were more likely to agree that both the school (mean = 3.94 ± 1.15) and the community that their children attended (mean = 3.90 ± 1.19) expected the children to receive COVID-19 vaccines. In comparison, the level of expectation from their spouse (mean = 3.34 ± 1.38), other family members (mean = 3.22 ± 1.30) and friends (mean = 3.11 ± 1.19) as the respondents perceived were lower. Only 25% of the respondents believed that their parents' friends would have their children take up the COVID-19 vaccination.

In terms of the construct *Perceived Behavioral Control*, the respondents rated the ease of arranging for their children to receive COVID-19 vaccines highly (mean = 3.93 ± 1.25). They were highly aware that the supply of COVID-19 vaccine in Macao was adequate (mean = 4.48 ± 0.87). Around 4.9% of the respondents agreed that it would be costly for their children to receive COVID-19 vaccines, and 6.5% of them indicated that they did not have the time to take their children for COVID-19 vaccination.

### Results of multiple linear regression

All factors found to be significantly associated with intention shown in Table 1 (including relationship with the child in question, age, number of children in total, age of child in question, perceived level of COVID-19 vaccine knowledge and personal history of COVID-19 vaccination) and Table 2 (the three TPB constructs) were analyzed as the independent variables, with intention as the dependent variable. Table 3 shows the results of multiple linear regression that analyzed the relationship between the intention of getting their children to receive COVID-19 vaccines and the independent variables.

**Table 2:** Measurement of the TPB constructs.

**Measures and sub-items of each measure**	**Mean**	**S/D**	**Frequency**										**Association of the construct subitem with Intention to have their children receive COVID-19 vaccination**		**Association of the construct with Intention to have their children receive COVID-19 vaccination**	
			**Strongly disagree**		**Disagree**		**Not sure**		**Agree**		**Strongly agree**			
			***n***	**%**	***n***	**%**	***n***	**%**	***n***	**%**	***n***	**%**	**Spearman's rho**	***P***	**Spearman's rho**	***P***
**Dependent variable—Intention**																
“*Based on my current knowledge about COVID-19 vaccines, I would give consent to vaccinating my child/children against COVID-19 infection.”*	3.29	±1.07	92	8	140	12	467	38	362	30	156	13				
**Independent variables—TPB constructs**																
**Construct 1—Attitude (Cronbach's alpha 0.881)**																
“*A COVID-19 vaccine will be important for my child/children's health.”*	3.45	±1.26	102	8.4	119	9.8	372	30.6	238	19.6	386	31.7	0.627**	0.000	0.718**	0.000
“*My child/children getting a COVID-19 vaccine would be a good way to protect him/her from COVID-19.”*	3.53	±1.28	123	10.1	113	9.3	345	28.3	273	22.4	363	29.8	0.653**	0.000		
“*A COVID-19 vaccine is effective if it is approved by the government.”*	3.26	±1.25	130	10.7	182	15.0	405	33.3	239	19.6	261	21.4	0.629**	0.000		
“*My child/children getting a COVID-19 vaccine will be important for the health of others in my community.”*	3.90	±1.15	52	4.3	92	7.6	289	23.7	281	23.1	503	41.3	0.603**	0.000		
“*A COVID-19 vaccine will not have been around long enough to be sure it is safe for children.”*	3.77	±1.22	84	6.9	80	6.6	336	27.6	254	20.9	463	38.0	−0.467**	0.000		
“*I am concerned about serious side effects of a COVID-19 vaccine for my child/children.”*	3.96	±1.23	71	5.8	86	7.1	251	20.6	216	17.7	593	48.7	−0.530**	0.000		
“*I think a COVID-19 vaccine might cause lasting health problems for my child/children.”*	3.54	±1.26	94	7.7	144	11.8	383	31.5	202	16.6	394	32.4	−0.497**	0.000		
**Construct 2—Subjective norm (Cronbach's alpha 0.888)**																
“*My spouse would support me in having my child/children take up COVID-19 vaccination.”*	3.34	±1.38	186	15.3	130	10.7	327	26.9	235	19.3	339	27.9	0.696**	0.000	0.724**	0.000
“*My other family members would support me in having my child/children take up COVID-19 vaccination.”*	3.22	±1.30	169	13.9	155	12.7	404	33.2	223	18.3	266	21.9	0.682**	0.000		
“*My friends would support me in having my child/children take up COVID-19 vaccination.”*	3.11	±1.19	152	12.5	147	12.1	536	44.0	180	14.8	202	16.6	0.646**	0.000		
“*Many of my parent friends will have their child/children take up COVID-19 vaccination.”*	2.88	±1.20	185	15.2	242	19.9	486	39.9	139	11.4	165	13.6	0.585**	0.000		
“*The school expects the students to take up COVID-19 vaccination.”*	3.94	±1.15	68	5.6	49	4.0	292	24.0	290	23.8	518	42.6	0.404**	0.000		
“*The community expects the children to take up COVID-19 vaccination.”*	3.90	±1.19	74	6.1	58	4.8	306	25.1	260	21.4	519	42.6	0.400**	0.000		
**Construct 3—Perceive behavioral control (Cronbach's alpha 0.516)**																
“*Having my child/children receive COVID-19 vaccination is easy for me if I want them to.”*	3.93	±1.25	90	7.4	71	5.8	247	20.3	241	19.8	568	46.7	0.332**	0.000	0.238**	0.000
“*Having my child/children receive COVID-19 vaccination is too costly for me financially.”*	1.47	±0.98	930	76.4	111	9.1	118	9.7	12	1.0	46	3.8	0.032	0.261		
“*I do not have time to take my child/children for COVID-19 vaccination.”*	1.64	±1.08	818	67.2	159	13.1	160	13.1	26	2.1	54	4.4	0.042	0.146		
“*I believe that there is an adequate supply of COVID-19 vaccine for children in Macao.”*	4.48	±0.87	19	1.6	18	1.5	140	11.5	220	18.1	820	67.4	0.194**	0.000		

**p < 0.01.

**Table 3:** Results of multiple regression analysis.

**Variables**	**Unstandardized coefficients**		**Standardized coefficients beta**	***t***	**Sig**.
	**B**	**Std. Error**			
**Model 1 (Control variables only)**					
(Constant)	2.93	0.27		10.739	0.000
**Control variables**					
Relationship with the child in question	−0.08	0.08	−0.030	−1.117	0.264
Age (years)	0.26	0.05	0.139	4.716	0.000
Number of children in total	0.07	0.04	0.047	1.718	0.086
Age of the child in question (years)	0.05	0.01	0.131	4.420	0.000
Perceived level of COVID-19 vaccine knowledge	0.07	0.03	0.055	2.060	0.040
No history of COVID-19 vaccination	−0.76	0.08	−0.259	−9.630	0.000
*F* = 34.053, d.f. = 6, *P* < 0.001, *R* = 0.380, *R*^2^ = 0.144, adjusted *R*^2^ = 0.140					
**Model 2 (TPB constructs, and control variables)**					
(Constant)	0.47	0.21		2.264	0.024
**Control variables**					
Age (years)	0.10	0.04	0.056	2.879	0.004
Age of the child in question (years)	0.01	0.01	0.017	0.892	0.373
Perceived level of COVID-19 vaccine knowledge	−0.01	0.02	−0.010	−0.573	0.567
No history of COVID-19 vaccination	−0.18	0.05	−0.063	−3.415	0.001
Number of children in total	−0.03	0.03	−0.020	−1.129	0.259
Relationship with the child in question	−0.03	0.05	−0.011	−0.612	0.541
**TPB constructs**					
TPB construct 1 - Attitude	0.52	0.03	0.457	17.738	0.000
TPB construct 2 - Subjective norm	0.39	0.03	0.363	14.070	0.000
TPB construct 3 - Perceived behavioral Control	0.02	0.04	0.012	0.668	0.505
*F* = 236.238, d.f. = 9, *P* < 0.001, *R* = 0.799, *R*^2^ = 0.638, adjusted *R*^2^ = 0.635					
**Model 3 (Sub-items of the TPB constructs, and control variables)**					
(Constant)	1.859	0.168		11.067	0.000
Age (years)	0.096	0.032	0.052	3.015	0.003
No history of COVID-19 vaccination	−0.206	0.053	−0.070	−3.886	0.000
**TPB construct 1 - Attitude**					
“*A COVID-19 vaccine will be important for my child/children's health.”*	0.067	0.024	0.078	2.758	0.006
“*Getting a COVID-19 vaccine would be a good way to protect child/children from COVID-19.”*	0.098	0.027	0.118	3.662	0.000
“*A COVID-19 vaccine is effective if it is approved by the government.”*	0.026	0.026	0.031	1.034	0.301
“*Getting a COVID19 vaccine will be important for the health of others in my community.”*	0.072	0.027	0.078	2.713	0.007
“*A COVID-19 vaccine will not have been around long enough to be sure it is safe.”*	−0.025	0.020	−0.029	−1.303	0.193
“*I am concerned about serious side effects of a COVID-19 vaccine.”*	−0.095	0.022	−0.109	−4.313	0.000
“*I think a COVID-19 vaccine might cause lasting health problems for my child/children.”*	−0.077	0.021	−0.091	−3.760	0.000
**TPB construct 2 - Subjective norm**					
“*My spouse would support me in having my child/children take up COVID-19 vaccination.”*	0.172	0.023	0.223	7.483	0.000
“*My other family members would support me in having my child/children take up COVID-19 vaccination.”*	0.090	0.028	0.109	3.261	0.001
“*My friends would support me in having my child/children take up COVID-19 vaccination.”*	0.051	0.029	0.056	1.755	0.079
“*Many of my parent friends will have their child/children take up COVID-19 vaccination.”*	0.089	0.024	0.100	3.736	0.000
“*The school expects the students to take up COVID-19 vaccination.”*	0.019	0.023	0.020	0.821	0.412
“*The community expects the children to take up COVID-19 vaccination.”*	−0.027	0.023	−0.030	−1.214	0.225
*F* = 156.628, d.f. = 15, *P* < 0.001, *R* = 0.813, *R*^2^ = 0.662, adjusted *R*^2^ = 0.658					

In Model 1, the independent variables comprised only the control variables, i.e., the demographic variables shown to have a statistically significant correlation with intention as shown in Table 1. The coefficients indicate that age of the respondents, the age of the child in question, the respondents' perceived level of COVID-19 knowledge and their personal history of COVID-19 vaccination had statistically significant influence over their intention to get their children vaccinated with COVID-19 vaccines. Nevertheless, Model 1 could only explain 14.4% of the variance in such (*R*^2^ = 0.144).

In Model 2, the control variables identified in Table 1 and the three TPB constructs indicated in Table 2 were computed as the independent variables. The coefficients indicated that *Attitude* and *Subjective Norm* as well as the respondents' personal history of COVID-19 vaccination and age were significant predictors of intention (all *p* < 0.001), whereas *Perceived Behavioral Control* and other demographic variables were not (all *p* > 0.05). In other words, only hypotheses H1 (Favorable *Attitude* is a positive and significant predictor of parents' intention to have their children receive COVID-19 vaccines) and H2 (positive *Subjective Norm* is a positive and significant predictor of parents' intention to have their children receive COVID-19 vaccines) are supported. A total of 63.7% (*R* = 0.799, adjusted *R*^2^ = 0.635) of the variance in the intention to get their children to receive the COVID-19 vaccine can be explained by Model 2 (*F* = 236.238, d.f. = 9, *p* < 0.001). Among the four statistically significant predictors, *Attitude* toward COVID-19 vaccination among children had stronger influence on their intention (β = 0.52, *p* < 0.001) as compared to that of *Subjective Norm* (β = 0.40, *p* < 0.001).

In Model 3, all the sub-items of *Attitude* and *Subjective Norm* as well as the respondents' personal history of COVID-19 vaccination and age were computed as the independent variables. The coefficients indicated that 5 out of 7 Attitude sub-items and 3 out of 6 *Subjective Norm* sub-items together with the respondents' personal history of COVID-19 vaccination and age were significant predictors of intention (all *p* < 0.001). Similar to Model 2, Model 3 can explain 65.8% (*R* = 0.813, adjusted *R*^2^ = 0.658) of the variance in the intention to get their children to receive the COVID-19 vaccine.

According to Model 3, in the order of the strongest influence, the positive predictors were: (1) *Subjective Norm* —“My spouse would support me in having my child/children take up COVID-19 vaccination.” (β = 0.223, *p* < 0.001); (2) *Attitude*—“My child/children getting a COVID-19 vaccine would be a good way to protect them from COVID-19.” (β = 0.118, *p* < 0.001); (3) *Subjective Norm*—“My other family members would support me in having my child/children take up COVID-19 vaccination.” (β = 0.109, *p* < 0.001); (4) *Subjective Norm*—“Many of my parent friends will have their child/children take up COVID-19 vaccination.” (β = 0.100, *p* < 0.001); (5) *Attitude*—“My child/children getting a COVID-19 vaccine will be important for my child/children's health.” (β = 0.118, *p* < 0.001); (6) *Attitude*—“My child/children getting a COVID19 vaccine will be important for the health of others in my community.” (β = 0.078, *p* < 0.001); and (7) older age (β = 0.052, *p* < 0.001). Negative predictors were: (1) *Attitude*—“I am concerned about serious side effects of a COVID-19 vaccine for my child/children.” (β = −0.109, *p* < 0.001); (2) *Attitude*—“I think a COVID-19 vaccine might cause lasting health problems for my child/children.” (β = −0.091, *p* < 0.001); and (3) No history of COVID-19 vaccination (β = −0.070, *p* < 0.001).

### Reasons for a lack of intention

**Figure 2:**
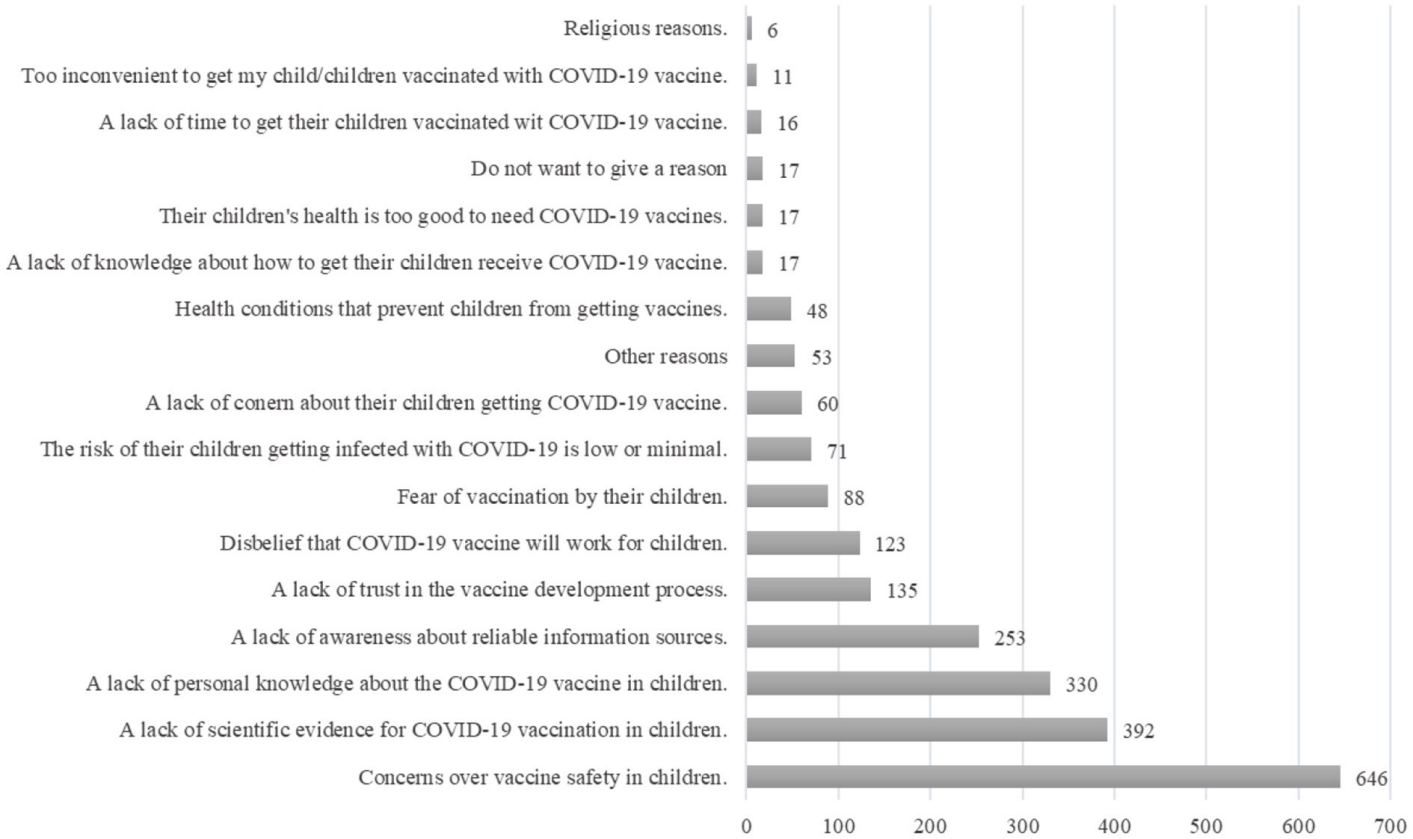
Reasons for not intending to get their children receive COVID-19 vaccines.

For respondents who indicated that they did not intend to get their children vaccinated with COVID-19 vaccines (*n* = 232) or were unsure (*n* = 467), they were asked to provide the reasons. As shown in Figure 2, a general concern over vaccine safety among children (*n* = 646/699, 92.4%) and a lack of scientific evidence for COVID-19 vaccination in children (*n* = 392/699, 56.1%) were cited as the most common reasons for their hesitation. This was followed by the awareness of personal shortcomings in terms of their knowledge about the use of COVID-19 vaccines in children (*n* = 330/699, 47.2%) and the lack of awareness about reliable information sources (*n* = 253/699, 36.2%). Some respondents also indicated a lack of trust in the vaccine development process (*n* = 135/699, 19.3%) or a disbelief that COVID-19 vaccine would work for children (*n* = 123/699, 17.6%).

Some respondents believed that the risk of their children getting infected with COVID-19 was low (*n* = 71/699, 10.2%) or that their children were too healthy to need COVID-19 vaccination (*n* = 17/699, 2.4%). A lack of knowledge about how to arrange their children to receive COVID-19 vaccines (*n* = 17/699, 2.4%), a lack of time to do so (*n* = 16/699, 2.3%), or a lack of convenience to make such an arrangement (*n* = 11/699, 1.6%) were also cited by some respondents. A small number of respondents cited health conditions (*n* = 48/699, 6.9%) or religious reasons (*n* = 6/699, 0.9%) as factors preventing their children from getting COVID-19 vaccines.

### Information sources considered reliable

The respondents were also asked to choose what they believed to be the reliable sources of information about COVID-19 vaccination among children. Doctors, among all the information sources and all the healthcare providers, were considered the reliable information sources by most respondents (*n* = 682). This was followed by international health organizations (e.g., the World Health Organization) (*n* = 526), the local government (*n* = 426) and the healthcare professional organizations (*n* = 416). News resources (such as newspapers, radio, and television) (*n* = 371) and online medical information (*n* = 313) were also cited as reliable information sources by many respondents. Some respondents also entrusted university academics (*n* = 195), pharmacists (*n* = 195) and nurses (*n* = 135) with the care of their patients. The schools which their children went were also considered reliable regarding information about the COVID-19 vaccine. It is also worth notice that social media (such as Facebook, Instagram, WeChat, Twitter, etc.) were also considered reliable by some respondents (*n* = 193).

## Discussion

This is one of the few studies that used the framework of the TPB to quantitatively examine parents' intention to have their children receive COVID-19 vaccines in areas with a low incidence rate. It was found that, 4 months into the COVID-19 vaccination program for children 3–12 years old in Macao, 42.5% of the parents indicated their intention, while 38.4% were not sure and 19.1% were negative. Parents' gender, age, number of children, perceived level of COVID-19 vaccine knowledge and history of COVID-19 vaccination, as well as the age of the child in question, demonstrated significant correlation with parents' intention. Importantly, it was also found that *Attitude* and *Subjective Norm but not Perceived Behavioral Control* were predictors of increased intention. Together with parents' age and history of COVID-19 vaccination, these predictors could explain 63.8% of the variance in parents' intention of vaccinating their children against COVID-19. Lack of intention was mostly due to the concerns about the vaccine safety and the lack of scientific evidence developed for children. Parents' decisions were also affected by their lack of vaccine knowledge and awareness of reliable information sources. In consistence with previous findings (52, 53), in helping parents make informed decision about vaccinating their children, healthcare providers, especially doctors, had a pivotal role to play.

The proportion of parents who intended to vaccinate their children against COVID-19 in Macao (42.5%) was lower than the overall proportion (60.1%, range 25.6–92.2%) as reported in a systematic review involving 43 studies from 18 countries (12). This is not surprising for areas like Macao, where the pandemic was under tight control and the number of infection cases was kept at a minimum. Uptake of COVID-19 vaccines by children was low due to low parental intention and was particularly challenging in areas that had been effective in stopping COVID-19 transmission. While increased perceived threat from the COVID-19 was related to parents' willingness to vaccinate their children against the COVID-19 (38, 47), vaccine complacency can be expected in the absence of perceived benefit from vaccination when there is no risk of infection as exemplified by the case of Macao (54).

The low perceived susceptibility may undermine the appreciation of the true benefits of the COVID-19 vaccine by parents, thus shifting their evaluation of the risk and benefit balance further toward safety concerns (39, 55, 56). Indeed, as reported in our study, some parents perceived the risk of their children contracting COVID-19 as low and considered the symptoms of the disease as mild; they reported less intent to have their children receive the vaccine (25). Studies have generally found that people who perceive COVID-19 to pose a greater risk engage more readily in preventive efforts (57, 58). It is obvious that some parents have yet to fully comprehend the possible impact of COVID-19 on children should a local widespread occur or a new variant emerge.

Another important factor affecting the parents' decision-making was their perceived risks of vaccination for their children (9, 42, 43, 59, 60). Out of the 699 parents who did not have the intention or were unsure about COVID-19 vaccination for their children, 646 quoted safety concerns as one of the main reasons. During the pandemic, some vaccine candidates had been granted fast-track licensure by the regulatory authorities, which contributed to vaccination hesitancy (40). Due to the short development process and the expedited approval procedures, evidence about the safety of the vaccines, especially among special age groups, was limited. Parents understandably expressed concern about the hurried nature of testing and expressed reservations about its safety (60). As such, it is crucial for the government and medical professionals to communicate effectively with parents about novel vaccine types and their safety evidence as it accumulates.

The concept of real-world evidence should also be introduced to the parents. For instance, while the results of a randomized, double-blind, controlled, phase 1/2 trial, which reported that the inactivated COVID-19 vaccine (the same type of COVID-19 vaccine available to children from 3 years in Macao) was safe and well tolerated in all participants aged 3–17 years, were published in early 2022 (61), 84 million children aged 3–11 in China had already received the first of two doses of the vaccine as of November 2021 (62). Similarly, in the US, 8 million of the ~28 million (28.1%) 5–11 year-old children had received at least one COVID-19 vaccine dose as of January 18, 2022, and the safety would be under close monitoring by the local public health authorities (63, 64). It is important for parents to be informed that while clinical trial data may be limited, the understanding of vaccine safety continues to grow with real world data. Parents were also worried about potential side effects, both immediate and long-term (65). Clarifying the side effects of COVID-19 vaccines may significantly reduce vaccination hesitancy.

The factors associated with parents' intention to vaccinate their children against COVID-19 aligned with most of the previous findings: father rather than mother (40, 41), older age (14, 66), older children (43, 44), high level of knowledge/information about the COVID-19 vaccination (33, 45), and COVID-19 vaccine uptake by the parents were associated with a higher intention to have their children receive vaccination (67, 68). These findings shed light on vaccination promotion strategies which should be targeted more specifically on mothers, younger parents, and those with younger children. Parents should be empowered to make vaccination decisions for their children with tailored and targeted communication materials and balanced information on both the benefits and risks of vaccination (69, 70). For this, it is important for the COVID-19 vaccine educational campaign implemented by public health officials to be robust and transparent.

Education about misinformation should also be prioritized as global misinformation spread through social media during the pandemic may pose challenges for COVID-19 vaccination programs (71). As shown in this study, social media was deemed reliable by many parents. However, previous research has already shown that there has been a lot of misinformation, conspiracy theories, and even anti-vaccine propaganda during the pandemic (72). The chance was that people might be more likely to bias toward negative information during a disease outbreak (73). The increasing influence of social media as a powerful tool for disseminating false data and unverified rumors, and the explosion of the available information made it difficult for parents to distinguish between true and false information (71). A unified and authoritative information outlet monitored by the local public health authority would be detrimental to preventing the creation of a vacuum with parents eager for advice and therefore turning to other sources to fill this informational void, which sometimes results in potential misinformation (67).

Who is responsible for delivering the education to achieve the best outcome should greatly depend on who the parents trust the most. In this study, most parents found doctors, international health organizations, and government and healthcare professional organizations reliable for advice about COVID-19 vaccination in children. Indeed, previous studies also stressed the importance of accessing scientific information or recommendations from public health authorities and physicians (15, 42, 74, 75). People who were exposed to scientific and positive information related to COVID-19 vaccines were shown to be more willing to have their children vaccinated (9, 41). In light of the above, the influence of stakeholders considered reliable by parents, such as doctors, health care professional organizations, or even schools as identified in this study, should be harnessed to reach out and raise people's awareness, to inform them of the importance of vaccines, and provide scientific information and recommendations on vaccinating their children against COVID-19 for parents from different walks of life (76).

The two important predictors of parents' intention to vaccinate their children against COVID-19 were *Subjective Norm* and *Attitude*. More specifically, it was the support and acknowledgment from their spouse, family, and friends; the perceived vaccine benefits for their children; and their sense of social responsibility that played the most part in encouraging their decision making. Previous studies have reported the positive influence of peer support (77, 78), confidence in COVID-19 vaccines (79), and perceived social responsibility on vaccination decision-making (80–82). Particularly in the context of Macao, the findings about 64.4% of parents agreeing to the importance of their children receiving the COVID-19 vaccine to the health of others in this study echoed previous findings about 67.8% of the general public considering getting the COVID-19 vaccination a social responsibility (83). This reinforced the importance of social norms on COVID-19 vaccination as emphasized by the World Health Organization that “*individuals and communities understand the value of vaccines and demand immunization as both their right and responsibility*” (84). Considering that promoting and displaying the social norms would help to shift public mentality toward health behavior change (85), publicly recognizing individual's social responsibility and empowering parents and their vaccinated children to share their experiences of and reasons for vaccination would help harness the power of social norm.

It is also worth discussing the reasons why *Perceived Behavioral Control* was not a predictor of parents' intention in this study. In particular, of the 4 sub-items under this construct, only the level of ease to make the decision and perceived vaccine supply were significantly associated with parents' intention, while cost and time were not. First, the COVID-19 vaccines were provided free-of-charge. Secondly, between February and May 2022, a series of actions to promote child vaccination have been initiated by the government to address different needs of parents. Schools were supported to organize in-house “vaccination days” operated by the outreach vaccination team. They were also recommended to continuously monitor parents' intentions and give them encouragement (86). A special “School Child Vaccination Day” was held to accommodate the parents' different work schedules. A mobile vaccination vehicle was also arranged to provide vaccination services to children and their parents who lived nearby (87). Representatives of pharmacist professional organizations were also invited to deliver public education directly to the parents at the schools (88). Such a multipronged campaign that includes education/promotion, good access to vaccines, and role models has helped to keep the cost or time constraints at a minimum, which were therefore not factored in parents' decision-making (67).

In Macao, as of May 7, 2022, more than 76% of children aged between 3 and 11 years had already received at least 1 dose of COVID-19 vaccines (87). In order to further promote children vaccination rate, as informed by the findings of this study, effective communication about the safety and the efficacy of the COVID-19 vaccine among children should remain prioritized in the vaccination campaign. For this, a number of actions should be considered: (1) While the public health authority continues to be the reliable sources of information about the safety and efficacy of COVID-19 vaccines, the government can also take the initiative to harness the trust of parents on healthcare professionals and have them play the role of “translators” to convey the scientific evidence in expressions that are easy to understand and reach out to different target parent groups; (2) Communication mechanisms should also make use of the close ties of schools and teachers with the parents to promote information sharing in their communities; (3) Collaboration between the public health authority and the research institutes should be reinforced to conduct studies on the immunogenicity, efficacy and safety of the COVID-19 vaccines among children based on local data; (4) Opportunities should be provided for parents of different demographic attributes to share their experiences and viewpoints on children vaccination to recognize and encourage the practice of social responsibility.

## Limitations

This study had a number of limitations. Firstly, under the impact of the pandemic, using an online platform to invite participants and operating the survey online were deemed the most feasible but might have induced sampling bias. It was not possible to find out the population to which the invitation was sent, so the response rate could not be determined. Also, due to a lack of demographic information about the overall parent population, the representativeness of the respondents could not be fully evaluated. Moreover, people who lacked technology literacy might be under-represented in this study. Secondly, due to the nature of a cross-sectional study, causal relationship between the key factors and the intention could not be inferred. As the factors affecting people's intention are subject to change as the pandemic evolves and the evidence about vaccine safety and efficacy emerges, findings from the current study may only provide a snapshot of the parents' intention around the beginning of the child vaccination program. Thirdly, the associations identified in this study mostly involved psychosocial factors. External factors, such as rewards of action, motivation to comply, and policy support, which might have an impact on *Attitude, Subjective Norm*, and *Perceived Behavioral Control*, respectively, were not considered in this study. Future studies are warranted to explore environmental factors that were not accounted for in this study but may play a role in predicting parents' intention to vaccinate their children against COVID-19 within the framework of TPB. As the vaccination requirements evolve over time, follow-up studies to monitor the changes in people's intentions to receive COVID-19 vaccination are warranted. In addition, as the topic of COVID-19 vaccination among children may be considered sensitive, the risks of social desirability bias cannot be ruled out which might have led the participants to indicate their indication when they in fact had not yet made the decision or had decided otherwise. Moreover, as this study was dependent upon voluntary participation, parents who found the topic of vaccinating their children sensitive or even controversial might not opt to take part in this study, resulting in non-response bias. Indeed, parents' with no intention to vaccinate their children in the study might have been under-represented in this study considering the differences between the proportion of parents with no intention to vaccinate their children with COVID-19 vaccines (19.1%) in this study and the non-vaccination rate of children aged 3 to up to 12 years old (< 25%) as of April 2022 according to official statistics. All the limitations mentioned above should be taken into consideration when interpreting the results of this study.

## Conclusions

This study found a moderate level of parents' intention to vaccinate their children against COVID-19, and empirically tested and demonstrated the utility of the TPB to evaluate how psychosocial attributes might guide the parents' decision-making. Targeted public health strategies should aim to address parents' concerns regarding COVID-19 vaccines and to provide precise and up-to-date information about vaccine uptake, safety, and effectiveness. Importantly, public awareness about the social responsibility associated with vaccination should be raised through orchestrated efforts of entities entrusted by parents. Continuous studies about the changes in the factors affecting parents' decision-making and the differences in the influences of such factors on various subgroups of parents would be instrumental for precise vaccination campaign design that achieves optimal children's uptake of a COVID-19 vaccine.

## Data availability statement

The raw data supporting the conclusions of this article will be made available by the authors, without undue reservation.

## Ethics statement

The studies involving human participants were reviewed and approved by University of Macau. The patients/participants provided their written informed consent to participate in this study.

## Author contributions

UC and YP carried out the literature review, collected and handled data, prepared data for analysis, performed statistics, interpreted the results, prepared the tables and figures, and drafted the manuscript. YZ and PT assisted in data analysis, interpreted the results, and reviewed the manuscript. HH supported data analysis, interpreted the results, and critically reviewed the manuscript. CU conceptualized and organized the study, carried out the literature review, confirmed and interpreted results, and critically reviewed and revised the manuscript. All authors contributed to the article and approved the submitted version.

## Funding

This research was funded by the University of Macau (SRG2021-00007-ICMS), the Key-Area Research and Development Program of Guangdong Province, China (2020B1111110003), Fund of University of Macau (EF013/ICMSWYT/2020/GDSTC), and the Science and Technology Development Fund, Macau SAR (SKL-QRCM(UM)-2020-2022).

## Conflict of interest

The authors declare that the research was conducted in the absence of any commercial or financial relationships that could be construed as a potential conflict of interest.

## Publisher's note

All claims expressed in this article are solely those of the authors and do not necessarily represent those of their affiliated organizations, or those of the publisher, the editors and the reviewers. Any product that may be evaluated in this article, or claim that may be made by its manufacturer, is not guaranteed or endorsed by the publisher.
