# Supplementary material for: Parents' intention for their children to receive COVID-19 vaccine: Implications for vaccination program in Macao
Source: Front Pediatr. 2022 Oct 3;10:978661. doi: 10.3389/fped.2022.978661 (PMC9575691; doi:10.3389/fped.2022.978661)
Supplement: Supplementary file 1 [file Data_Sheet_1.docx]

**Parents' intention for their children to receive COVID-19 vaccine: a survey study**

**PARTICIPANT INFORMATION STATEMENT**

1. What is this study about?

This study aims to investigate the acceptance of Macau's parents for free immunization of COVID-19 for children under 18 years of age and to explore possible enablers to support such practice.

The coronavirus disease 2019 (COVID-19) pandemic continues to develop around the world without any signs of subsiding any time soon. On a global scale, children and adolescents under 20 years of age account for 16% of the reported COVID19 cases and 33% of the population; among the COVID-19 deaths, 0.4% (over 11,700) occur in children and adolescents under 20 years of age. Children are beginning to be vaccinated, and the public health department in increasing number of countries and regions has called for the expansion of vaccination coverage to include children. Therefore, it is important to understand their parents' vaccination attitude and tendency to act.

Using Macao as a case study, we aim to seek your insights on the issue of vaccinating your child against COVID-19.

You have been invited to participate in this study because you are a parent or a legal guardian of children under 18 and live in Macau in the past 12 months.

The Participant Information Statement tells you about the research study. Knowing what is involved will help you decide if you want to take part in the research. Please read this sheet carefully and ask questions about anything that you do not understand or want to know more about.

By completing and submitting the survey, we assume that you:

(1) understand the Participant Information Statement

(2) agree to take part in the research study as outlined in the Participant Information Statement

(3) agree to the use of their personal information collected in the survey, and

(4) are providing consent to take part in the study.

1. Who is running the study?

The study has been granted ethics approval by The Panel on Research Ethics, University of Macau and is being carried out by the following researcher: Dr Carolina UNG Assistant Professor, Institute of Chinese Medical Sciences, University of Macau Department of Public Health and Medicinal Administration, Faculty of Health Sciences, University of Macau

1. What will the study involve for me?

To be a survey participant, you will be invited to complete a survey on online hosted by the online survey distribution company Survey Monkey and can be accessed through the web link https://www.surveymonkey.com/r/Parent_KidCovid19Vaccine_Macao.

1. How much time will the survey take?

The survey will take approximately 10 minutes.

1. Do I have to be in the study?

Participating in this survey is completely voluntary and you do not have to take part. Your participation is completely anonymous

1. Can I withdraw from the study?

Before submitting the survey, you can withdraw from the study or change any information you have provided in the survey at any-time. However, after the survey has been submitted, it is not possible for you to withdraw from this study or to change any information you have provided in the survey. This is because your participation is completely anonymous and any data collected from the submitted survey is unidentifiable. Therefore, once the survey has been completed and submitted, you participation cannot be withdrawn and the information you have provided cannot be changed.

1. Are there any risks or costs associated with being in the study?

Aside from giving up your time, we do not expect any risks or costs associated with taking part in this study.

1. Are there any benefits associated with being in the study?

By participating in this survey you are contributing to developing the body of knowledge that will inform the current acceptance of Macau's parents for free COVID-19 vaccination for children under 18 years of age. However, there will be no other benefits associated with your support and participation.

1. What will happen to data I provide?

The data collected will be stored securely and you cannot be identified from your survey responses. The results will be kept strictly confidential, except as required by law. Study findings may be published or presented, but you will not be individually identifiable in these publications or presentations. Electronic files will be kept in a laptop with a key-in password only the lead researcher knows. All data will be retained for five years after the study and then all the electronic files will be removed from the hard drive and memory card permanently and all the hard copies of information, if any, will be shredded before disposal.

1. Can I tell other people about the study?

Yes, you are welcome to tell other people about the study.

1. What if I would like further information about the study?

When you have read this information, Dr Carolina Ung will be available to discuss it with you further and answer any questions you may have. If you would like to know more at any stage during the study, please feel free to contact Dr Ung by phone (+853-8822-4672) or email [carolinaung@um.edu.mo](mailto:carolinaung@um.edu.mo).

**This PARTICIPANT INFORMATION STATEMENT is for you to keep.**
